# High expression of miR-17-5p and miR-20a-5p predicts favorable disease-specific survival in stage I-III colon cancer

**DOI:** 10.1038/s41598-022-11090-2

**Published:** 2022-04-30

**Authors:** Hallgeir Selven, Sigve Andersen, Mona I. Pedersen, Ana Paola Giometti Lombardi, Lill-Tove Rasmussen Busund, Thomas Karsten Kilvær

**Affiliations:** 1 Department of Oncology, University Hospital of North Norway, 9038 Tromso, Norway; 2 Department of Clinical Medicine, UiT The Arctic University of Norway, Tromso, Norway; 3 Department of Medical Biology, UiT The Arctic University of Norway, Tromso, Norway; 4 Department of Clinical Pathology, University Hospital of North Norway, Tromso, Norway

**Keywords:** Cancer, Gastrointestinal cancer, Tumour biomarkers

## Abstract

In many types of cancer, microRNAs (miRs) are aberrantly expressed. The aim of this study was to explore the prognostic impact of miR-17-5p and miR-20a-5p in colon cancer. Tumor tissue from 452 stage I-III colon cancer patients was retrospectively collected and tissue microarrays constructed. miR-17-5p and miR-20a-5p expression was evaluated by in situ hybridization and analyzed using digital pathology. Cell line experiments, using HT-29 and CACO-2, were performed to assess the effect of miR-17-5p and miR-20a-5p over expression on viability, invasion and migration. In multivariate analyses, high miR-17-5p expression in tumor (HR = 0.43, CI 0.26–0.71, *p* < 0.001) and high expression of miR-20a-5p in tumor (HR = 0.60, CI 0.37–0.97, *p* = 0.037) and stroma (HR = 0.63, CI 0.42–0.95, *p* = 0.027) remained independent predictors of improved disease-specific survival. In cell lines, over expression of both miRs resulted in mitigated migration without any significant effect on viability or invasion. In conclusion, in stage I-III colon cancer, high expression of both miR-17-5p and miR-20a-5p are independent predictors of favorable prognosis.

## Introduction

Colon cancer is the 4th most and 5th most common cause of cancer and cancer related deaths, respectively^1^. In 2020, it is estimated that 1 150 000 patients experienced a de novo colon cancer and that 575 000 succumbed to their disease. Despite improved diagnostics and treatment, and decreasing incidence, the mortality of colon cancer remains high. To further tailor the treatment of these patients, the development of novel prognostic and predictive biomarkers is important^2,3^.

MicroRNAs (miRs or miRNAs) are short, non-coding RNAs, approximately 22 nucleotides long. They regulate gene expression at the post-transcriptional level. An estimated 30% of human genes are regulated by miRs^4^. miRs influence diverse biological mechanisms including apoptosis, growth, differentiation and proliferation^5^. In cancer, miRs act as both oncogenes and tumor suppressors^6^. The miR-17 ~ 92 cluster, located at chromosomal locus 13q31.3, comprises six tandem stem-loop hairpin structures that yield six mature miRs (miR-17, miR-18a, miR-19a, miR-19b, miR-20a and miR-92a)^7^. There are two miR-17 ~ 92 cluster paralogs in mammals: miR-106b ~ 25 located on chromosome 7 and miR-106a ~ 363 located on the X-chromosome, comprising an additional 6 and 3 mature miRs. Collectively, these 15 mature miRs are grouped into four families, namely the miR-17, miR-18, miR-19 and miR-92^8^. The polycistronic structure of miR cluster genes differs from most protein coding genes, as multiple miRs can be produced within a single pri-miR transcript. Each of these can act independently^9^. miR-17 ~ 92 is predominantly related to cell cycle regulation. In normal development it is involved in lung and heart maturation and hematopoiesis, where it promotes cell proliferation and survival^10^. The cluster was first discovered in 2005, when it was found to act with c-MYC to promote tumorigenesis in B-cell lymphomas^11^. Over expression of the miR-17 ~ 92 cluster has been observed in multiple tumor types including hematological malignancies (B-cell lymphomas) and solid tumors (breast, lung, CRC, pancreas and prostate)^11,12^.

The miR-17 family comprise miRs 17 and 20a in the miR-17 ~ 92 cluster, 106a and 20b in the miR-106a ~ 363 cluster and 106b and 93 in the miR-106b ~ 25 cluster^8^. Hence, miR-17 and miR-20a are the only members of the miR-17 family situated in the miR-17 ~ 92 cluster. Of interest, the expression level of mature miRs belonging to the same cluster are not equivalent^13^. An early discovery was that c-MYC activates expression of the miR-17 ~ 92 cluster by binding directly to its locus on chromosome 13. c-MYC also targets transcription factor E2F1, promoting cell cycle progression. E2F1 is negatively regulated by both miR-17 and miR-20a. Thus, on one side c-MYC activates members of the E2F family of transcription factors, and on the other side limits their translation^14^. Subsequent studies identified that miR-17-5p and miR-20a-5p expression is suppressed by p53 and NKX3.1, stimulated by MXI1 and STAT, and that their expression suppress known regulators of cell death, cell cycle regulation, hypoxia, angiogenesis, and proliferation^15^. In gastrointestinal cancers (gastric, CRC and HCC), miR-17 has been related to increased cell proliferation, migration and invasion and reduced overall survival^16^. In contrast, miR-17 was shown to act as a tumor suppressor in breast-, cervical- and prostate cancer^17–19^. miR-20a regulates cell proliferation and cancer progression, and is dysregulated in both solid and hematopoietic cancers^15^. miR-20a was related to poor survival in lung- and gastric cancer, among others^20,21^. Whereas in oral squamous cell carcinoma and hepatocellular carcinoma, miR-20a acted as a tumor suppressor^22,23^.

Previous studies in gastrointestinal cancers reported that both miR-17 and miR-20a frequently are over expressed and that miR-17 was associated with an unfavorable prognosis^16,24^. However, to our knowledge these studies included patients with advanced disease, combined separate entities such as colon and rectal adenocarcinoma and did not distinguish between expression in tumor epithelial cells and tumor stroma. Therefore, we explore the prognostic impact of miR-17-5p and miR-20a-5p expression in tissue from colon cancer patients treated with curative intent. We supplement our results with functional experiments in select early-stage colon cancer cell lines. And hypothesize that these miRs are clinically relevant biomarkers in localized colon cancer.

## Results

### Patient characteristics

The patient characteristics have previously been reported^25^. Briefly, 452 patients were included in the study. There was a minor female predominance (53.8% vs 46.2%), and the median age at surgery was 74 years (range 30–94). Seventy-two (15.9%), 219 (48.5%) and 161 (35.6%) patients were diagnosed with pTNM stage I-III, respectively. Median follow-up of survivors was 173 months. At the end of follow-up, 119 patients had recurrent disease and 313 patients were dead, either due to colon cancer (108) or other causes (205).

### Expression of miR-17-5p and miR-20a-5p and their correlations

**Figure 1:**
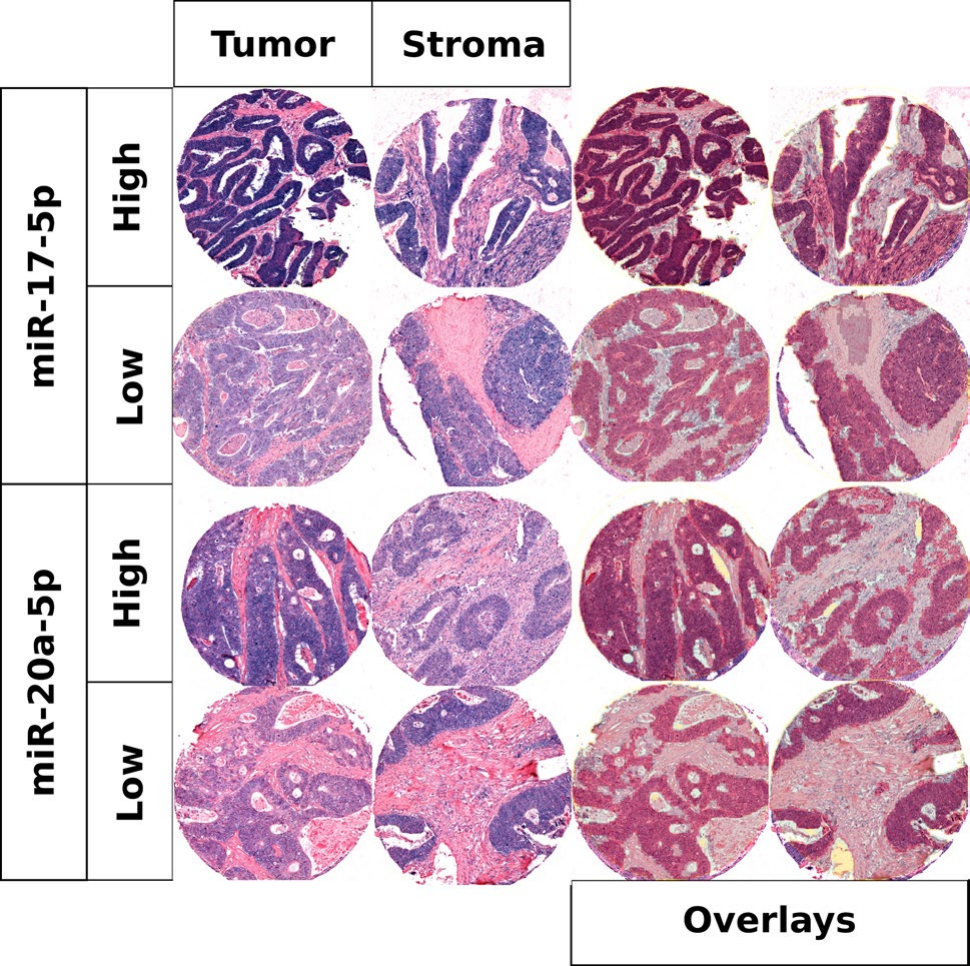
High and low scores for miR-17-5p and miR-20a-5p in tumor and stroma with and without overlays.

miR-17-5p and miR-20a-5p were mostly expressed in tumor epithelial cells and to a varying degree in stromal cells including spindle shaped cells (likely fibroblasts, endothelial cells and vascular smooth muscle cells) and immune cells (Fig. 1).

Correlations between miRs and clinicopathological variables are presented in Table 1. High expression of both miR-17-5p and miR-20a-5p in tumor tissue was associated with well and moderately differentiated tumors. In addition, miR-20a-5p in tumor was associated with cancers of the right colon. Between-miR correlations were as follows: miR-17-5p in tumor was correlated with mir-17-5p in stroma (r = 0.27), miR-20a-5p in tumor (r = 0.52) and stroma (r = 0.17); miR-17-5p in stroma was correlated to miR-20a-5p in tumor (r = 0.16) and stroma (r = 0.37); miR-20a-5p in tumor was correlated with miR-20a-5p in stroma (r = 0.65).

**Table 1:** Dichotomized miR-17-5p and miR-20a-5p in tumor and stroma and their distribution over and correlation with clinicopathological variables (chi-square and Fisher’s exact tests).

	miR-17-5p in tumor			miR-17-5p in stroma			miR-20a-5p in tumor			miR-20a-5p in stroma		
	Low	High	*p*	Low	High	*p*	Low	High	*p*	Low	High	*p*
**Age**			0.115			0.947			0.742			0.472
≤ 65	80	27		26	83		76	31		27	80	
> 65	213	110		83	252		244	89		72	266	
**Gender**			0.547			1.000			0.930			0.665
Female	154	77		59	181		170	65		51	189	
Male	139	60		50	154		150	55		48	157	
**Weight_loss**			**0.027**			0.818			0.055			0.507
< 10%	151	81		62	180		172	71		52	194	
≥ 10%	70	19		22	71		73	16		23	68	
**ECOG_status**			0.827			0.093			0.526			0.066
0	150	76		47	185		163	68		45	188	
1	100	41		45	102		106	38		42	104	
2	35	16		14	39		42	11		9	45	
3	6	2		3	5		7	1		3	5	
**Site**			0.101			0.133			**0.023**			0.150
Sigmoid	158	58		48	177		165	56		40	183	
Transversum	42	20		16	49		53	11		17	48	
Left	12	6		3	16		16	5		7	14	
Right	79	52		42	90		83	48		34	99	
**pStage**			0.085			**0.006**			0.306			0.165
1	42	28		10	61		45	24		10	60	
2	139	70		48	168		160	57		49	170	
3	112	39		51	106		115	39		40	116	
**Hist_grade**			**0.015**			0.463			**0.026**			0.722
Well	22	13		10	26		20	14		6	29	
Moderate	203	111		81	240		230	93		74	251	
Poor	59	12		14	61		61	12		15	59	
Undifferentiated	3	1		0	4		3	0		1	2	
**Vasc + **			0.281			0.323			0.131			0.066
No	128	60		49	146		149	45		43	152	
Yes	14	3		7	11		17	1		8	10	
**Resection margins**			0.234			0.255			0.571			0.609
0 mm	25	4		12	19		24	5		6	25	
< 1 mm	31	10		9	33		31	11		9	33	
1-2 mm	22	11		6	29		22	13		4	31	
2-10 mm	76	38		28	89		82	35		26	91	
10-50 mm	94	52		34	117		111	38		39	113	
> 50 mm	30	15		15	31		33	13		11	35	

*ECOG*, Eastern cooperative oncology group, *pStage*, pathological stage, *Hist_grade*, histological grade, *Vasc + *, vascular infiltration.

Statistically significant values in bold.

### Cell line experiments

**Figure 2:**
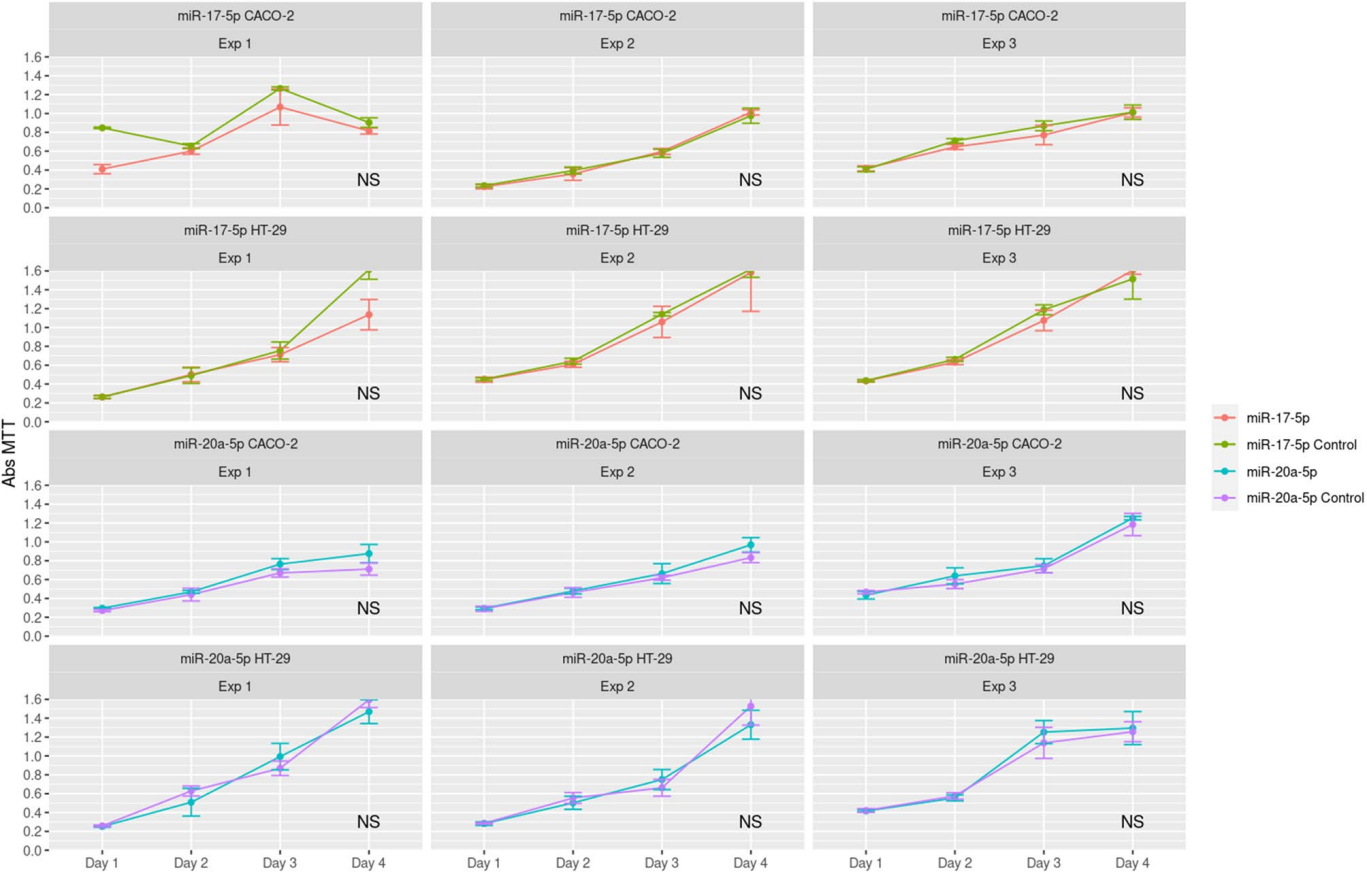
MTT assays comparing viability in cells transfected with either miR-17-5p (rows 1 and 2) or miR-20a-5p (rows 3 and 4) with control in the CACO-2 (rows 1 and 3) and HT-29 (rows 2 and 4) cell lines.

**Figure 3:**
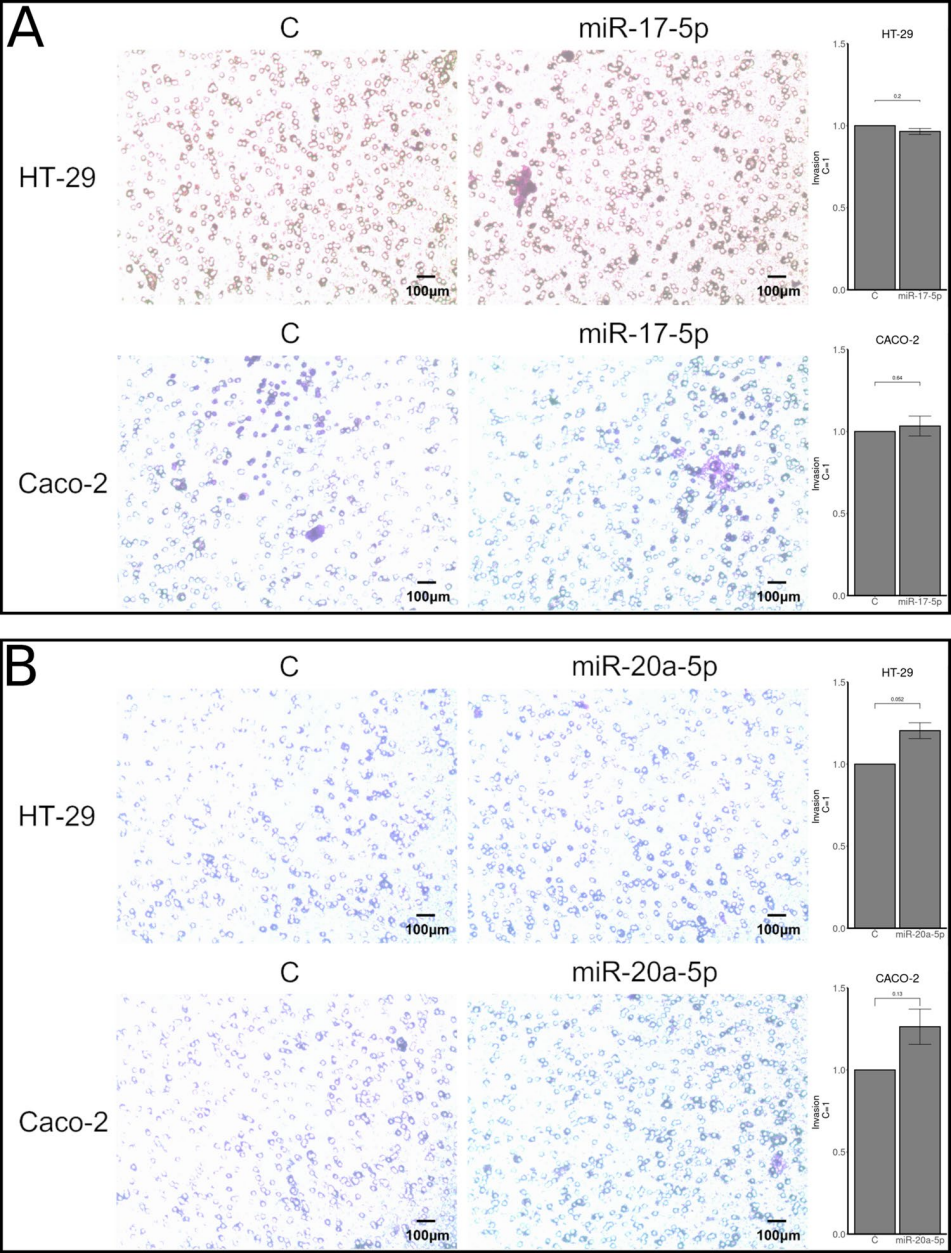
Transwell assays as measures of invasion/migration in CACO-2 and HT-29 cell lines transfected with miR-17-5p (panel A) or miR-20a-5p (panel B). Results are plotted as a mean of 3 experiments + /− SEM and relative to control (C = 1).

**Figure 4:**
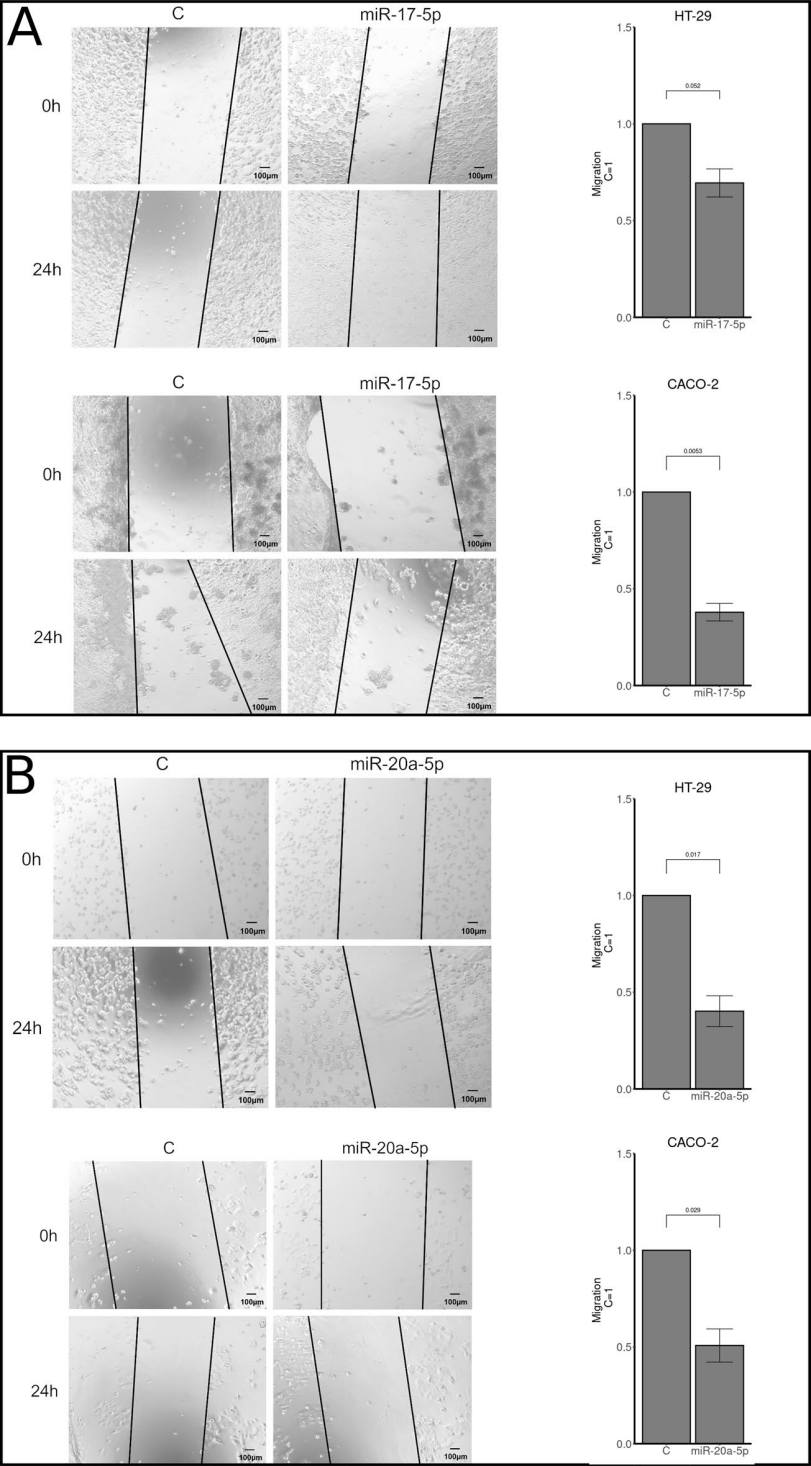
Wound healing assays as measures of migration in CACO-2 and HT-29 cell lines transfected with miR-17-5p (panel A) or miR-20a-5p (panel B). Results are plotted as a mean of 3 experiments + /− SEM and relative to control (C = 1).

HT-29 and CACO-2 cell lines were tested for viability using MTT assays and invasion using transwell assays. No differences in viability or invasion were observed when either miR-17-5p or miR-20a-5p was over expressed compared to controls (Figs. 2 and 3). For migration analyses, using wound healing assays, reduced migration rates after miR-17-5p and miR-20a-5p over expression was observed for both cell lines (Fig. 4). These findings were statistically significant for both miR-17-5p (p < 0.001) and miR-20a-5p (p = 0.029) in the CACO-2 cell line. For the HT-29 cell line, the results for miR-20a-5p were statistically significant (p = 0.017) and borderline significant for miR-17-5p (p = 0.052).

### Univariate analyses

**Figure 5:**
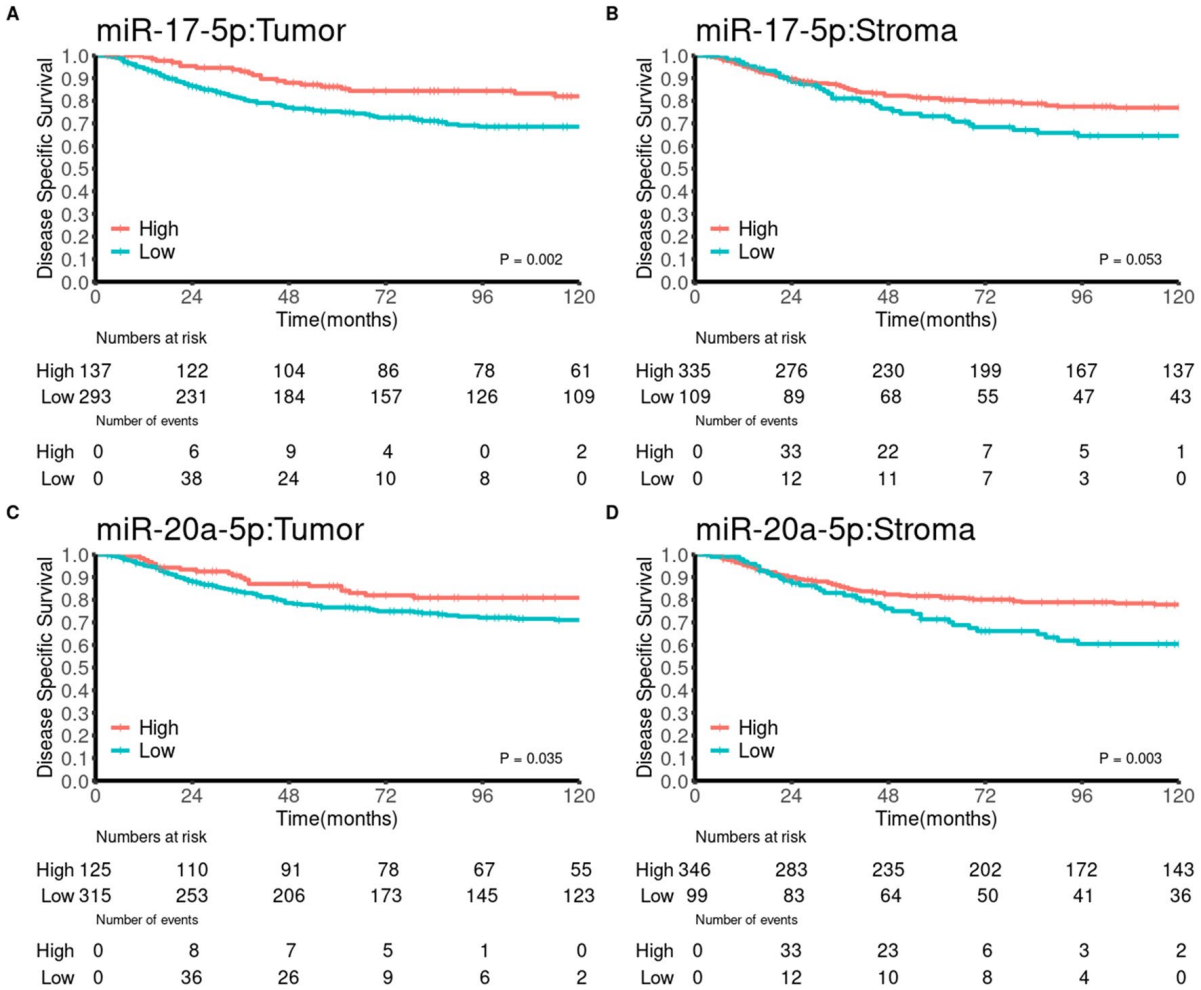
Disease-specific survival curves for expression of miR-17-5p and miR-20a-5p using the optimal cut-offs for each marker.

Univariate survival analyses of clinicopathological variables were presented previously^25^. In brief, age, weight loss, pathological stage, histological grade, vascular infiltration, and resection margins were significant indicators of DSS. Univariate analyses of the investigated markers are presented in Table 2 and Fig. 5. High miR-17-5p expression in tumor was a significant indicator of DSS (p = 0.002), while high miR-20a-5p expression was a significant indicator of DSS in tumor (p = 0.035) and stroma (p = 0.003).

**Table 2:** Univariate analyses of miR-17-5p and miR-20a-5p in tumor and stroma (log-rank, n = 452).

	N	5Y	M	HR (95% CI)	*p*
**miR-17-5p in tumor**					**0.002**
Low	293(65)	75	NA	1.000	
High	137(30)	86	NA	0.48(0.32–0.72)	
Missing	22(5)				
**miR-17-5p in stroma**					0.053
Low	109(24)	73	NA	1.000	
High	335(74)	81	NA	0.67(0.42–1.05)	
Missing	8(2)				
**miR-20a-5p in tumor**					**0.035**
Low	315(70)	77	NA	1.000	
High	125(28)	86	NA	0.6(0.39–0.92)	
Missing	12(3)				
**miR-20a-5p in stroma**					**0.003**
Low	99(22)	71	NA	1.000	
High	346(77)	82	NA	0.55(0.35–0.88)	
Missing	7(2)				

*N*, number, *5Y*, 5-year survival, *M*, median survival, *p*, *p*-value, *HR*, hazard ratio, *CI*, confidence interval, *NA*, not applicable.

Statistically significant values in bold.

### Multivariate analyses

Multivariate analyses are summarized in Table 3. All significant variables from the univariate analyses were entered into the initial analyses. In the final models, high miR-17-5p expression in tumor was independently associated with favorable DSS (HR = 0.43, CI 0.26–0.71, p < 0.001), while high miR-20a-5p expression remained an independent predictor of favorable DSS in both tumor and stroma, (HR = 0.60, CI 0.37–0.97, p = 0.037) and (HR = 0.63, CI 0.42–0.95, p = 0.027), respectively.

**Table 3:** Multivariate models for miR-17-5p in tumor (I) and for miR-20a-5p in tumor (II) and stroma (III), (cox proportional hazards tests, n = 452).

	I		II		III	
	HR (95% CI)	*p*	HR (95% CI)	*p*	HR (95% CI)	*p*
**miR-17-5p in tumor**						
Low	1					
High	0.43(0.26–0.71)	** < 0.001**				
Missing						
**miR-20a-5p in tumor**						
Low			1			
High			0.60(0.37–0.97)	**0.037**		
Missing						
**miR-20a-5p in stroma**						
Low					1	
High					0.63(0.42–0.95)	**0.027**
Missing						
Age at diagnosis	1.03(1.01–1.05)	**0.002**	1.03(1.01–1.05)	**0.009**	1.02(1.01–1.04)	**0.013**
**Differentiation**						
Well differentiated	1					
Moderately differentiated	0.91(0.42–2.01)	0.822				
Poorly differentiated	0.61(0.25–1.51)	0.286				
Undifferentiated	7.97(1.93–32.95)	**0.004**				
**pTNM**						
pTNM-stage I	1		1		1	
pTNM-stage II	2.32(0.89–6.03)	0.085	2.18(0.85–5.58)	0.105	2.16(0.84–5.55)	0.108
pTNM-stage III	8.27(3.26–21.01)	** < 0.001**	7.85(3.16–19.55)	** < 0.001**	7.58(3.04–18.88)	** < 0.001**

*p*, *p*-value, *HR*, hazard ratio, *CI*, confidence interval, *pTNM*, pathological tumor-node-metastasis.

Statistically significant values in bold.

## Discussion

We present results showing favorable prognosis for colon cancer patients with high expression of miR-17-5p or miR-20a-5p. Patients with high expression of miR-17-5p in tumor tissue had a 5-year DSS of 86% compared with 75% for low expression. Patients with high expression of miR-20a-5p in tumor and stromal tissue had a 5-year DSS of 86% and 82% *vs* 77% and 71% for low expression, respectively (Table 2 and Fig. 1). Our results diverge from previous findings in similar cohorts and highlights important considerations for future studies on miRs in gastrointestinal cancer patients.

Contrasting our findings, most previous studies in gastrointestinal cancers related high miR-17-5p expression to impaired prognosis^26^. However, the number of high-quality studies exploring the prognostic value of miR-17-5p expression in localized colon cancer is limited. A meta-analysis from 2018, investigated the prognostic impact of miR-17-5p expression in gastrointestinal cancers^16^. Pooled analyses suggested that high expression of miR-17-5p predicted both poor overall survival (HR = 1.86, CI 1.55–2.25, p < 0.001) and poor disease-free survival (HR = 1.43, CI 1.01–2.03, p = 0.046). Of interest, ~ half of the identified publications reported non-significant results and/or were based on miR expression in serum/plasma, all studies included patients with advanced disease and several distinct cancers were represented^27^. Similar to our study, three studies report the prognostic impact of miR-17-5p expression in tissue from CRC patients^27–29^. Contrary to our study, these studies included stage I-IV patients, used overall survival as endpoint and did not distinguish between colon and rectal cancer. Both Ma and Fang et al*.* reported that high levels of miR-17-5p in tumor tissue, identified using ISH, was associated with impaired survival in Asian CRC patients^28,29^. Diaz et al*.* did not observe a survival difference in European patients^27^. This latter observation may suggest a demographic difference in CRC patients as proposed by others^30^. Moreover, although all three studies reported a similar percentage of miR-17-5p high patients, both Ma and Fang et al*.* observed increased miR-17-5p expression in stage III and IV patients^28,29^. Similar to our study, Diaz et al. observed a decline in miR-17-5p expression with increasing stage^27^. These results further corroborate the notion of a demographic difference for these biomarkers. In addition, neither Ma and Fang nor Diaz properly address the potential confounder introduced with patients with metastatic disease or rectal cancer.

Several previous studies stated that miR-20a-5p is upregulated both in feces and tumor tissue from colon cancer patients ^12,31,32^. In our study, we observed that high expression of miR-20a-5p, both in tumor and stromal tissue, was related to a favorable disease-specific survival. Our findings contradict a recent meta-analysis assessing the efficacy of miR-20a as a diagnostic and prognostic biomarker for colorectal cancer^24^. The meta-analysis, comprised of thirty-two studies, six including colon cancer patients only, concluded that miR-20a-5p expression was associated with impaired overall survival. However, similar to studies in miR-17-5p, several issues including differences in methodology, patient demographics and study endpoints precludes direct comparison with our study. Of interest, Signs et al*.* explored the impact of miR-20a-5p expression in the stromal compartment of colitis-associated cancer. They observed that stromal miR-20a-5p expression was higher in normal colon compared to a colitic or cancerous colon. Further, low levels of miR-20a-5p correlated with low levels of the inflammatory and oncogenic chemokine CXCL8 secreted by stromal fibroblasts. Stromal downregulation of miR-20a expression appeared to occur prior to epithelial upregulation. This suggests that downregulated miR-20a-5p expression in fibroblasts in the colitic field is responsible for the upregulation of CXCL8 responsible for tumorigenesis in colitis-associated cancer^33^. These findings are in line with our results, where high stromal expression of miR-20a-5p correlates to better outcome for the patients.

To further elucidate the role of miR-17-5p and miR-20a-5p, we investigated their functional aspects in two colon cancer cell lines. HT-29 and CACO-2 are known to form low-grade/early-stage cancer when grown in nude mice and are thus likely representative of localized colon cancer^34,35^. Interestingly, over expression of the miRs did not impact viability or invasion and mitigated migration in both cell lines. These results strengthen our findings in patients with localized colon cancer, where over expression of miR-17-5p and miR-20a-5p predicted better outcome for the patients. Corroborating our findings, several groups observed that over expression and/or suppression of miR-17-5p and miR-20a-5p subsequently mitigated and promoted migration in colon cancer cells^36,37^. Of particular interest, Ast et al*.* investigated the role of miR-17-5p and tumor-stromal cell interaction in the setting of CRC carcinogenesis^38^. By co-culturing colon cancer cells and colon fibroblasts transfected with a miR-17 mimic, they noticed significantly reduced cell invasion. Increased expression of miR-17 also significantly reduced the invasive activity of fibroblasts. However, other groups report that over expression of miR-17-5p and miR-20a-5p increases proliferation, migration and invasion in colon cancer cell lines, thus highlighting the complex role of these miRs in distinct settings^28,29,39^.

The conflicting results hamper both interest and implementation of use of miR-17-5p and miR-20a-5p as biomarkers in colon cancer. Nevertheless, according to clinicaltrials.gov, Wu Song and co-workers are validating a signature of six miRs to predict chemotherapy response in stage II colon cancer^40^. This trial is based on their previous work^41^, but no results are as of yet presented from their trial. Regardless of the outcome from the ongoing validation by Wu Song, our data indicate that positive results need to be validated and not automatically extrapolated to other demographic groups.

## Conclusion

In conclusion, we have shown that high expression of miR-17-5p in tumor tissue and high expression of miR-20a-5p in both tumor and stroma are independent indicators of favorable disease-specific survival for localized colon cancer. Our findings contradict previous studies in colorectal cancer, and highlights that potential differences in methodology, patient demographics and endpoints may highly influence the prognostic value of these biomarkers. Further, although data from several pre-clinical studies and our cell line studies corroborates our findings, contrasting results exists also in this domain. Due to these contradictions, prospective trials resolving these issues have to be conducted before clinical implementation of miR-17-5p and miR-20a-5p as prognostic or predictive biomarkers in colon cancer can be considered.

## Materials and methods

### Study population

Patients who underwent radical surgery for colon cancer, in various hospitals in Northern Norway from 1998–2007, were eligible for inclusion. Initially, 861 patients were identified. Of these, 409 patients were excluded, mainly due to metastatic disease/prior malignancy within the last 5 years before diagnosis, missing tissue blocks/inadequate tissue for TMA construction or miscoding (mainly rectal cancer). Hence, 452 patients were included in the study. Follow-up was completed December 1, 2017. Detailed information about the study population was previously published^25^.

### Tissue Microarray construction

All colon cancer cases were reviewed by two pathologists, and the most representative areas of tumor without necrosis were selected. A 0.6 mm-diameter stylet was used to sample a total of 4 cores securing both tumor and stroma from each included patient. The TMAs were assembled using a tissue-arraying instrument (Beecher Instruments, Silver Springs, MD, USA). The detailed methodology has previously been reported^42^.

### In situ hybridization (ISH)

The microRNA in situ hybridization method was performed on the Ventana Discovery Ultra platform for IHC and ISH. The protocol was developed by Roche, (Tucson, USA), based on the manual protocol previously published by Jorgensen et al*.*^43^.

Double‐DIG labeled miRCURY LNA detection probes and control probes from Exiqon (Exiqon AS, Denmark) was used to define the expression level of miR-20a-5p and miR-17-5p in colon cancer FFPE tissue. Detection kits and buffers purchased from Roche gave the chromogenic visualization of the microRNAs.

Slides were baked at 60 °C overnight, and then transferred to the Discovery Ultra for ISH staining. Sections were deparaffinized at 68 °C for three cycles in Ventana EZ buffer. Heat retrieval was performed at 95 °C with Discovery Cell Conditioning Solution (CC1) for 40 min to make access for the probes. Optimized concentrations of probe controls and target miR probes were manually applicated, miR-20a-5p, 50 nM, and miR-17-5p, 20 nM. The hybridization reaction was carried out for 60 min at 54 °C for miR-17-5p and 40 °C for miR-20a-5p followed by two stringency washes with 2.0X SSC buffer. Possible unspecific bindings were blocked with AB blocking solution for 16 min. Alkaline phosphatase-conjugated anti DIG (Anti-DIG-AP) was incubated for 20 min for immunologic detection. Substrate enzymatic reactions were carried out with NBT/BCIP for 60–120 min to give a blue precipitate. The slides were counterstained with Nuclear Fast Red for contrast staining. Slides were dehydrated through an increasing gradient of ethanol solutions to xylene and mounted with Histokitt mounting medium.

Good sensitivity level of the ISH method and minimal RNA degradation in tissue was confirmed by U6, snRNA control probe at a concentration of 1.5 nM. 10 nM scramble miR negative control indicated no unspecific staining from reagents or tissues. The level of microRNA expression in other tissues than colon cancer was confirmed by a TMA multi tissue control. Optimizations regarding temperatures, times, and concentrations were done for each probe and reagent.

### In situ hybridization scoring/QuPath

The details of the digital workflow is described thoroughly in our previous paper^25^. In brief, TMA slides were digitized and processed in QuPath v.0.1.3 according to Bankhead et al*.*^44^. Tissue within each TMA core was identified and tiled. Image features were used to train a Random Forest model. Each tile was classified as either tumor, stroma, necrosis or other. After classification, tiles were converted into continuous areas and the mean intensity of miR-17-5p and miR-20a-5p within tumor and stroma was calculated. The scripts used to process the TMAs are included in the supplementary file.

All possible dichotomized cut-offs were evaluated. For any subsequent analyses, the optimal cut-off was chosen.

### Cell line experiments

#### Cell cultures

The functional aspects of miR-17-5p and miR-20a-5p were tested in two colon cancer cell lines: CACO-2 (ATCC HTB-37) and HT-29 (ATCC HTB-38) both derived from colon adenocarcinoma. They have been authenticated and recently tested negative for mycoplasma contamination. The cell lines were tested for viability, migration and invasion in the absence and presence of miR-17-5p and miR-20a-5p as previously described by Stoen et al*.*^45,46^. The most important steps of each assay are referred below.

#### Cell culture and transfection

Cells were transiently transfected with either 10 µM has-miR-17-5p Pre-miR miRNA Precursor (catalog# PM12412, Thermo Fisher Scientific, USA) or has-miR-20a-5p Pre-miR miRNA Precursor (catalog# AM17100, Thermo Fisher Scientific, USA), alongside the Cy3 Dye-Labeled Pre-miR Negative Control #1 (catalog# AM17120, Thermo Fisher Scientific, USA) using the transfection reagent Lipofectamine RNAiMAX (catalog#13,778,075, Thermo Fisher Scientific, USA). Transfected Cy3 Dye-Labeled Pre-miR Negative Control emits fluorescent light when exposed to UV-light, and using a fluorescence microscope, the transfection efficiency was estimated to 80–95%.

### Viability assay

Cells were cultured in 96-well plates and incubated with 12 mM of [3-(4,5-dimethylthiazol-2-yl)-2,5-diphenyltetrazolium bromide] (MTT, 5 mg/ml) (cat.# M6494, Invitrogen, OR, USA). Formazan crystals were solubilized by addition of 0,01 M HCl/SDS (cat.# 28,312, Thermo Scientific, IL, USA) and the absorbance was measured in the CLARIOstar plate reader (BMG Labtech, Ortenberg, Germany) at 570 nm.

### Migration/wound healing assay

Cells were grown in a 24-well plate, washed with PBS and incubated in a serum free medium with mitomycin C (10 µg/L) to avoid cell proliferation. The cells were “wounded” using a 200 µl sterile pipette tip and then washed to remove detached cells and debris. After 4 h the cells were transfected. To measure wound healing in controls and transfected cells, photographs of the same areas of the wound were taken at 0 and 24 h. Images were captured using a Nikon Eclipse TS100 inverted optical microscope and analyzed by Micrometrics SE Premium 4 software. Areas occupied by migrating cells after 24 h were calculated by subtracting the background levels at 0 h.

### Invasion assay

Cells were seeded in ThincertR chambers (Greiner Bio-one, Kremsmünster, Austria) with polyethylene terephthalate membranes (8 mm pore size) pre-coated with 50 mL of phenol red-free Matrigel (Gibco). These chambers were placed in 24-well plates containing culture medium with 10% FBS in the lower chamber. Cells in the upper chambers were transfected and incubated for 48 h at 37 °C. The chambers were washed thoroughly with 10 mM PBS, fixed in 4% paraformaldehyde for 30 min, and stained with 0.2% crystal violet for 10 min. Non-invading cells, from the membrane upper surface, were removed using a cotton swab. The membranes containing the invaded cells (under the membrane surface), were photographed. Images of three random microscope fields were captured in duplicate, using an inverted optical microscope Nikon Eclipse TS100. The areas of cell invasion were determined by Image J software.

### Statistical methods

Statistical tests were performed in Rstudio 2021.09.0 build 351 (RStudio PBC) using R version 4.0.4. DSS (disease-specific survival) was defined as the interval from surgery to the time of colon cancer death. Before analyses, expression of all miRs were rescaled to a range between 0 and 1 using max–min scaling. For univariate analyses, the Kaplan–Meier method was used to visualize associations between molecular marker expression and survival. The log-rank test was used to assess the statistical significance of the differences between the survival curves. Multivariate analyses were performed using a backward conditional Cox regression analysis with a probability for stepwise entry and removal at 0.05 and 0.10, respectively. A p-value < 0.05 was considered statistically significant.

### Ethics declaration

The study was conducted according to the guidelines of the Declaration of Helsinki, and was approved by the Regional Committee for Medical and Health Research Ethics North (REK Nord, protocol ID: 2011/2151). The need for informed consent was waived by REK Nord due to the retrospective nature of the study. The reporting of clinicopathological variables, survival data and biomarker expression was conducted in accordance with the REMARK guidelines^47^.

## Supplementary Information

Supplementary Information.

## Data Availability

Data will be shared upon reasonable request to the corresponding author.
