# Supplementary material for: High expression of miR-17-5p and miR-20a-5p predicts favorable disease-specific survival in stage I-III colon cancer
Source: Sci Rep. 2022 Apr 30;12:7080. doi: 10.1038/s41598-022-11090-2 (PMC9056518; doi:10.1038/s41598-022-11090-2)
Supplement: Supplementary file 1 — Supplementary Information. [file 41598_2022_11090_MOESM1_ESM.pdf]

```
//Script for miR-17-5p

import qupath.lib.objects.PathAnnotationObject
import qupath.lib.objects.TMACoreObject
import qupath.lib.objects.hierarchy.PathObjectHierarchy

setImageType('BRIGHTFIELD_OTHER');
setColorDeconvolutionStains('{"Name" : "miR17_Red", "Stain 1" : "miR17", "Values 1" : "0.71029 0.65126 0.26711 ", "Stain 2" : "Red", "Values 2" : "0.14996 0.89274 0.42488 ", "Background" : " 255 255 255 "}');
selectTMACores();
runPlugin('qupath.imagej.detect.tissue.SimpleTissueDetection2', '{"threshold": 249,  "requestedPixelSizeMicrons": 2.0,  "minAreaMicrons": 10000.0,  "maxHoleAreaMicrons": 1000.0,  "darkBackground": false,  "smoothImage": true,  "medianCleanup": true,  "dilateBoundaries": false,  "smoothCoordinates": true,  "excludeOnBoundary": false,  "singleAnnotation": true}');
selectAnnotations();
runPlugin('qupath.lib.algorithms.TilerPlugin', '{"tileSizeMicrons": 10.0,  "trimToROI": true,  "makeAnnotations": false,  "removeParentAnnotation": false}');
selectDetections();
runPlugin('qupath.lib.algorithms.IntensityFeaturesPlugin', '{"pixelSizeMicrons": 2.0,  "region": "ROI",  "tileSizeMicrons": 25.0,  "colorOD": true,  "colorStain1": true,  "colorStain2": true,  "colorStain3": false,  "colorRed": true,  "colorGreen": true,  "colorBlue": true,  "colorHue": true,  "colorSaturation": true,  "colorBrightness": true,  "doMean": true,  "doStdDev": true,  "doMinMax": true,  "doMedian": true,  "doHaralick": true,  "haralickDistance": 1,  "haralickBins": 32}');
selectTMACores();
runPlugin('qupath.lib.plugins.objects.SmoothFeaturesPlugin', '{"fwhmMicrons": 25.0,  "smoothWithinClasses": false,  "useLegacyNames": false}');
runPlugin('qupath.lib.plugins.objects.SmoothFeaturesPlugin', '{"fwhmMicrons": 75.0,  "smoothWithinClasses": false,  "useLegacyNames": false}');
removeObjects(getAnnotationObjects(), true)

runClassifier('path_to_your_classifier’)
selectTMACores();
runPlugin('qupath.lib.analysis.objects.TileClassificationsToAnnotationsPlugin', '{"pathClass": "All classes",  "deleteTiles": true,  "clearAnnotations": false,  "splitAnnotations": false}');
selectAnnotations();
runPlugin('qupath.lib.algorithms.IntensityFeaturesPlugin', '{"pixelSizeMicrons": 2.0,  "region": "ROI",  "tileSizeMicrons": 25.0,  "colorOD": false,  "colorStain1": true,  "colorStain2": false,  "colorStain3": false,  "colorRed": false,  "colorGreen": false,  "colorBlue": false,  "colorHue": false,  "colorSaturation": false,  "colorBrightness": false,  "doMean": true,  "doStdDev": true,  "doMinMax": true,  "doMedian": true,  "doHaralick": false,  "haralickDistance": 1,  "haralickBins": 32}');

def addMeasurements(PathObjectHierarchy hierarchy, TMACoreObject core, pixelWidth, pixelHeight) {
    def annotations = hierarchy.getDescendantObjects(core, null, PathAnnotationObject)

    //Check if core has annotation
    if (annotations.size() > 0) {

        //check if each annotation is of interest, and if so, keep track of the positive and negative cells within
```

```
        annotations.each { annotation ->
                annotationName = annotation.getPathClass().getName()
            if (annotation.getROI().getScaledArea(pixelWidth, pixelHeight) >
MIN_AREA_MICRONS) {
                def measures = annotation.getMeasurementList()
                measures.each {
                    def measureName = it.getName()tokenize()[5,6].join()
                    def measure = it.getValue()
                    core.getMeasurementList().putMeasurement("${annotationName} ${measureName}",
measure)
                }
            }
        }
    }else{
    core.setMissing(true)
    }
}
MIN_AREA_MICRONS = 40000
def server = getCurrentImageData().getServer()
double pixelWidth = server.getPixelWidthMicrons()
double pixelHeight = server.getPixelHeightMicrons()
def hierarchy = getCurrentHierarchy()
def cores = hierarchy.getTMAGrid().getTMACoreList()
cores.each {
    addMeasurements(hierarchy, it, pixelWidth, pixelHeight)
}
fireHierarchyUpdate()
println("Finished")
```

```
//Script for miR-20a-5p

import qupath.lib.objects.PathAnnotationObject
import qupath.lib.objects.TMACoreObject
import qupath.lib.objects.hierarchy.PathObjectHierarchy

setImageType('BRIGHTFIELD_OTHER');
setColorDeconvolutionStains('{"Name" : "miR20a_colon", "Stain 1" : "miR20a-5p", "Values 1" :
"0.66521 0.71006 0.2309 ", "Stain 2" : "Red", "Values 2" : "0.22275 0.86345 0.45259 ",
"Background" : " 255 255 255 "}');
selectTMACores();
runPlugin('qupath.imagej.detect.tissue.SimpleTissueDetection2', '{"threshold": 250,
"requestedPixelSizeMicrons": 2.0,  "minAreaMicrons": 1000.0,  "maxHoleAreaMicrons":
100000.0,  "darkBackground": false,  "smoothImage": true,  "medianCleanup": true,
"dilateBoundaries": false,  "smoothCoordinates": true,  "excludeOnBoundary": false,
"singleAnnotation": true}');
selectAnnotations();
runPlugin('qupath.lib.algorithms.TilerPlugin', '{"tileSizeMicrons": 10.0,  "trimToROI": true,
"makeAnnotations": false,  "removeParentAnnotation": false}');
selectDetections();
runPlugin('qupath.lib.algorithms.IntensityFeaturesPlugin', '{"pixelSizeMicrons": 2.0,  "region":
"ROI",  "tileSizeMicrons": 10.0,  "colorOD": true,  "colorStain1": true,  "colorStain2": true,
"colorStain3": false,  "colorRed": true,  "colorGreen": false,  "colorBlue": true,  "colorHue": true,
"colorSaturation": true,  "colorBrightness": true,  "doMean": false,  "doStdDev": true,
"doMinMax": false,  "doMedian": true,  "doHaralick": true,  "haralickDistance": 1,  "haralickBins":
32}');
selectAnnotations();
runPlugin('qupath.lib.plugins.objects.SmoothFeaturesPlugin', '{"fwhmMicrons": 25.0,
"smoothWithinClasses": false,  "useLegacyNames": false}');
runPlugin('qupath.lib.plugins.objects.SmoothFeaturesPlugin', '{"fwhmMicrons": 75.0,
"smoothWithinClasses": false,  "useLegacyNames": false}');
removeObjects(getAnnotationObjects(), true)


runClassifier('path_to_your_classifier’);
selectTMACores();
runPlugin('qupath.lib.analysis.objects.TileClassificationsToAnnotationsPlugin', '{"pathClass": "All
classes",  "deleteTiles": true,  "clearAnnotations": false,  "splitAnnotations": false}');
selectAnnotations();
runPlugin('qupath.lib.algorithms.IntensityFeaturesPlugin', '{"pixelSizeMicrons": 2.0,  "region":
"ROI",  "tileSizeMicrons": 25.0,  "colorOD": false,  "colorStain1": true,  "colorStain2": false,
"colorStain3": false,  "colorRed": false,  "colorGreen": false,  "colorBlue": false,  "colorHue": false,
"colorSaturation": false,  "colorBrightness": false,  "doMean": true,  "doStdDev": true,
"doMinMax": true,  "doMedian": true,  "doHaralick": false,  "haralickDistance": 1,  "haralickBins":
32}');

def addMeasurements(PathObjectHierarchy hierarchy, TMACoreObject core, pixelWidth,
pixelHeight) {
    def annotations = hierarchy.getDescendantObjects(core, null, PathAnnotationObject)

    //Check if core has annotation
    if (annotations.size() > 0) {
```

```
        //check if each annotation is of interest, and if so, keep track of the positive and negative cells
within
        annotations.each { annotation ->
                annotationName = annotation.getPathClass().getName()
            if (annotation.getROI().getScaledArea(pixelWidth, pixelHeight) >
MIN_AREA_MICRONS) {
                def measures = annotation.getMeasurementList()
                measures.each {
                    def measureName = it.getName()tokenize()[5,6].join()
                    def measure = it.getValue()
                    core.getMeasurementList().putMeasurement("${annotationName} ${measureName}",
measure)
                }
            }
        }
    }else{
    core.setMissing(true)
    }
}
MIN_AREA_MICRONS = 40000
def server = getCurrentImageData().getServer()
double pixelWidth = server.getPixelWidthMicrons()
double pixelHeight = server.getPixelHeightMicrons()
def hierarchy = getCurrentHierarchy()
def cores = hierarchy.getTMAGrid().getTMACoreList()
cores.each {
    addMeasurements(hierarchy, it, pixelWidth, pixelHeight)
}
fireHierarchyUpdate()
println("Finished")
```
